# *Saccharomyces boulardii* Improves Intestinal Cell Restitution through Activation of the α2β1 Integrin Collagen Receptor

**DOI:** 10.1371/journal.pone.0018427

**Published:** 2011-03-31

**Authors:** Alexandra Canonici, Carole Siret, Emilie Pellegrino, Rodolphe Pontier-Bres, Laurent Pouyet, Marie Pierre Montero, Carole Colin, Dorota Czerucka, Véronique Rigot, Frédéric André

**Affiliations:** 1 Inserm, UMR 911, Centre de Recherche en Oncologie et Oncopharmacologie, Marseille, France; 2 Aix-Marseille Université, UMR 911, Marseille, France; 3 Team 4: Inflammation, Cancer, Cancer stem cells, INSERM U895, Centre Méditerranéen de Médecine Moléculaire (C3M), Nice, France; 4 Inserm U 624, Marseille, France; Emory University, United States of America

## Abstract

Intestinal epithelial cell damage is frequently seen in the mucosal lesions of inflammatory bowel diseases such as ulcerative colitis or Crohn's disease. Complete remission of these diseases requires both the cessation of inflammation and the migration of enterocytes to repair the damaged epithelium. Lyophilized *Saccharomyces boulardii (Sb*, Biocodex*)* is a nonpathogenic yeast widely used as a therapeutic agent for the treatment and prevention of diarrhea and other gastrointestinal disorders. In this study, we determined whether *Sb* could accelerate enterocyte migration. Cell migration was determined in *Sb* force-fed C57BL6J mice and in an *in vitro* wound model. The impact on α2β1 integrin activity was assessed using adhesion assays and the analysis of α2β1 mediated signaling pathways both *in vitro* and *in vivo*. We demonstrated that *Sb* secretes compounds that enhance the migration of enterocytes independently of cell proliferation. This enhanced migration was associated with the ability of *Sb* to favor cell-extracellular matrix interaction. Indeed, the yeast activates α2β1 integrin collagen receptors. This leads to an increase in tyrosine phosphorylation of cytoplasmic molecules, including focal adhesion kinase and paxillin, involved in the integrin signaling pathway. These changes are associated with the reorganization of focal adhesion structures. In conclusion *Sb* secretes motogenic factors that enhance cell restitution through the dynamic regulation of α2β1 integrin activity. This could be of major importance in the development of novel therapies targeting diseases characterized by severe mucosal injury, such as inflammatory and infectious bowel diseases.

## Introduction

The colonic epithelium forms a continuous physical and functional barrier that protects the internal environment of the body from the fluctuating external milieu. Various substances including dietary elements, gastrointestinal secretory products and drugs are known to disrupt this epithelial barrier, leading to the shedding of epithelial cells and the development of wounds [1]. In addition inflammatory bowel diseases (IBD), including ulcerative colitis and Crohn's disease, are characterized by varying degrees of mucosal surface damage, chronic inflammation and mucosal ulceration, resulting in the subsequent impairment of the barrier function [2]. Moreover, infection of the colonic mucosa by bacterial pathogens including *Shigella*, *Salmonella* or *Clostridium difficile* results in the development of acute intestinal inflammatory diseases and destruction of the intestinal epithelium [3], [4]. The colonic barrier has a striking ability to rapidly reseal superficial wounds, which is critical for the maintenance of barrier function and homeostasis. As with other epithelia of the gastrointestinal tract, the repair of damaged colonic mucosa initially requires cell migration to restore epithelial continuity [1]. This process, termed restitution, is followed by the proliferation and subsequent maturation and differentiation of the cells, allowing the restoration of normal architecture and absorptive/secretory function.

Intestinal restitution has been found to be influenced by a broad spectrum of factors derived from the gastrointestinal environment, including host epithelial and lamina propria cells, resident microbiota, and the dietary and non-dietary components present in the gastrointestinal lumen [2]. Moreover, dynamic and reciprocal crosstalk between receptors for soluble factors and those for the extracellular matrix (ECM) play a crucial role in the regulation of this process [5], [6], [7].

Integrins constitute the main cell surface adhesion receptors mediating cell-ECM adhesion. These transmembrane heterodimeric molecules are made up of non-covalently bound α and β subunits. In mammals, 18 α and 8 β subunits combine to form 24 distinct integrin receptors that bind various ECM ligands with different affinities [8]. Integrins allow a bi-directional flow of mechanochemical information across the plasma membrane and facilitate interactions between the ECM and the actin cytoskeleton. These integrin-mediated interactions, on either side of the plasma membrane, are dynamically linked. The cytoskeleton controls the functional state of the integrins thus modulating their interaction with the ECM. Meanwhile integrin binding to the ECM changes the cell shape and the composition of the cytoskeleton beneath [8].

The nonpathogenic yeast *Saccharomyces boulardii* (*Sb*) is widely used in a lyophilized form to treat and prevent antibiotic-associated and infectious diarrhea [9]. Recent *in vitro* and *in vivo* studies indicate that *Sb* interacts not only with pathogenic micro-organisms and resident microflora, but also with intestinal mucosa [10], [11]. In addition *Sb* has been shown to exert a trophic effect that restores intestinal homeostasis [11]. Furthermore, clinical trials have suggested that *Sb* can be effective in the treatment of IBD [12], [13] through the modulation of host cell signaling pathways implicated in the proinflammatory response [14], [15], [16]. However, no information is currently available on the possible effects of *Sb* upon colonic epithelial cell restitution.

In the present study, we show that *Sb* secretes factors that modulate intestinal epithelial cell restitution both *in vitro* and *in vivo*. Our findings suggest that *Sb* increases intestinal epithelial cell migration without affecting cell proliferation. *Sb* exerts at least some of its motogenic effects through the activation of the α2β1 integrin collagen receptor signaling pathway, which is associated with the reorganization of focal adhesions.

## Methods

### Cell culture

The human colonic adenocarcinoma cell lines HCT-8/E11, CaCo2/TC7, HT29-D4 and T84, were routinely cultured as previously described [17], [18], [19], [20]. Cells were cultured on plastic dishes until they reached confluency. These cellular monolayers consist of polarized cells joined by tight junctions, exhibiting well developed apical microvilli, which allow the study of the processes involved in intestinal epithelial cell physiology.

### Yeast culture supernatants

The supernatants of *Sb* (Biocodex laboratories; Gentilly, France) and *Saccharomyces cerevisiae* (*Sc*, strain BY4742, a gift from C. De La Roche Saint-André; CNRS, Marseille, France) were prepared as previously described [21]. Briefly, yeast strains (100 mg/ml) were cultured overnight at 37°C in epithelial cell culture media. Conditioned media were centrifuged at 20,000× *g* for 15 minutes and the supernatants collected. The supernatants were passed through 0.22 µm filters (Fisher Scientific) to remove yeast cells. Serial dilutions ranging from 1/8 to 1/128 were performed in epithelial cell culture media. None of the diluted supernatants affected cell viability, as verified by the trypan blue exclusion test.

### Wound healing model

Monolayers of differentiated cells were wounded using a sterile tooth-pick and incubated with or without various dilutions of *Sb* supernatant. Plates were placed in a temperature and CO_2_-controlled chamber mounted on a Nikon TE2000 inverted microscope. Images were captured every 5 minutes for a total observation period of 5 hours using a Cool SnapHQ camera (Princeton Instruments) through a 10× objective lens. For each wound, 10 measurements of wound width were recorded. To assess the role of the integrins in *Sb* enhanced enterocyte migration, 10 µg/ml function-blocking anti-integrin monoclonal antibodies (mAbs) (GoH3 (anti-α6), 69.6.5 (anti-αv), Lia1/2 (anti-β1), Gi9 (anti-α2), and SAM-1 (anti-α5); Beckman Coulter, Marseille, France), β1-activating mAb (TS2/16; Santa Cruz Biotechnology, Santa Cruz, CA, USA) or anti DPP IV mAb (S. Maroux, CNRS, Marseille) were added 1 hour before wounding and again during the period of cell migration. In some experiments, MAPK inhibitor (10 µM PD 98059; Tocris Bioscience, Bristol, UK ) or 10 nM FAK inhibitor (PF 573228; Tocris Bioscience) were added 1 hour before wounding and again during the period of cell migration. When required, cells were treated with 2.5 µg/mL mitomycin C (Sigma) prior and during cell migration in order to block cell proliferation. In order to track the rate of cell proliferation, 10 µmol/L 5-bromo-2′-deoxyuridine (BrdU) was added 4 hours after the wound and incubation continued for an additional 1 hour period. Cells that had incorporated BrdU into their DNA were detected using the BrdU labeling and detection kit according to the manufacturer's instructions (Roche, Mannheim, Germany). Cell proliferation in wounded monolayers was visualized by light microscopy using an inverted Olympus CKX41 microscope.

### Measurement of enterocyte crypt-villus migration

Groups of 6-week old C57BL6J female mice (n = 5) were force-fed daily with 200 µL PBS solution with or without 1 mg/ml lyophilized *Sb* for 1 week. When required, mice were force-fed daily for one week with 200 µL *Sb* supernatant or *Sc* supernatant. Animals were injected with BrdU (50 mg/kg, BrdU; Sigma) intraperitoneally and then sacrificed 24 hours later. Segments of intestine were frozen in liquid nitrogen and cryosectioning (section thickness: 8 µm) was performed. Samples were immunostained with a rat anti-BrdU antibody mAb (Abcam) overnight at 4°C. After washing, sections were incubated with AlexaFluor 488-conjugated goat anti-rat IgG (Invitrogen) for 1 hour, and then mounted in ProLong Gold medium containing DAPI (Invitrogen) in order to couterstain nuclei. Images were captured and analyzed using a Leica DM IRBE microscope. Enterocyte migration was determined by measuring the distance from the bottom of the crypt to the foremost labeled enterocyte and expressing the distance as a percentage of the total villus height. All animal experiments were performed in accordance with the regulations of our institution's ethics commission. They were conducted following the APS *Guiding Principles in the Care and Use of Animals*. The study was approved by the Ethics Committee in Animal Experimentation of Centre Méditerranéen de Médecine Moléculaire (C3M), Nice, France (protocol 3/2010).

### Cell adhesion assay

Cell adhesion assays on Laminin-111 (Sigma, Saint Quentin Fallavier, France) and type I collagen (BD-Bioscience, Belford, MA, USA) were performed using cells incubated with or without *Sb* supernatant, as previously published [22]. Function blocking anti-integrin mAbs (10 µg/mL) were added to a subset of samples, to identify the receptor types involved in *Sb* enhanced adhesion.

### Immunocytochemistry and Immunohistochemistry

Cells, incubated with or without *Sb* supernatant, were fixed and permeabilized as previously described [23]. Paxillin was immunostained with a mouse anti-paxillin mAb (Millipore). Tissues were fixed in acetone for 10 minutes at -20°C, and stained with rat anti-ki-67 (Abcam), rat anti-α6 integrin (Beckman coulter), rabbit anti-α2 integrin (Millipore) or rabbit anti-Y118 Paxillin (Millipore) Abs. After washing, samples were incubated with the appropriate Alexa 488- or Alexa 546-conjugated secondary Abs (Invitrogen), and then mounted in ProLong Gold medium. Images were captured and analyzed using a Leica DM IRBE microscope.

### Detection of tyrosine-phosphorylated proteins

Single-cell suspensions (25 000 cells/0.1 mL), prepared in DMEM containing 0.2% BSA (adhesion buffer), were seeded onto type I collagen-coated wells and allowed to adhere for periods ranging from 0 to 30 minutes. Adherent cells were lyzed as previously published [24]. Equal amounts of cell lysates (50 µg) were resolved by SDS-PAGE and blotted onto a nitrocellulose sheet. Membranes were blocked with PBS containing 5% non-fat dry milk and probed overnight at 4°C with Abs directed against rabbit phospho ERK1/2 (Ozyme, St. Quentin en Yvelines, France), mouse anti-Y397-FAK (Invitrogen) or rabbit anti-Y118-paxillin (Millipore). Blots were then revealed by chemiluminescence after incubation with the appropriate horseradish peroxidase-conjugated secondary Ab (Amersham). Loading amounts were verified by probing the blot with rabbit-anti FAK (Ozyme), mouse anti-Erk1/Erk2 (Santa Cruz Biotechnology), or mouse anti-paxillin (Millipore).

### Statistical analysis

All experiments were performed a minimum of 3 times in duplicate. For statistical analysis of data, Student's t-test was used. Values are expressed as mean ± SD. Data were considered as statistically significant at *P* values of <.01(**) or <.05 (*).

## Results

### *Sb* supernatant improves colonic epithelial cell restitution

**Figure 1:**
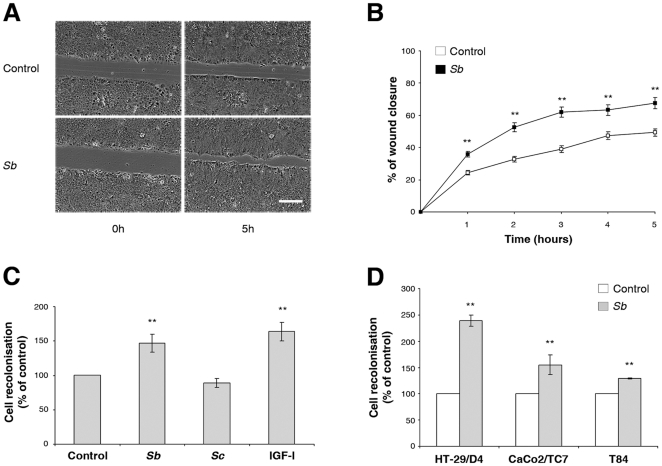
*Sb* increases intestinal epithelial cell restitution. (**A**) Polarized HCT-8/E11 cell monolayers were wounded as described in Methods and incubated for 5 hours with or without *Sb* supernatant. Phase contrast images were acquired at the indicated times (see also Supplementary data S**1** and S**2**). Data shown are from a representative experiment out of 5 performed. Scale bar: 300 µm. (**B**) HCT-8/E11 cell monolayers were wounded and incubated with or without *Sb* supernatant. The percentage of wound area closed was determined at hourly intervals. Data represent the mean ± SD of 3 separate experiments. ** *P*<.01. (**C**) HCT-8/E11 cell monolayers were wounded and incubated for 5 hours without (control) or with *Sb* or *Sc* supernatants, or 14 nM IGF-I (IGF-I). Results are expressed as the percentage of cell migration compared to control. Data represent the mean ± SD of 3 separate experiments. ** *P*<.01. (**D**) Polarized HT29-D4, CaCo2/TC7 and T84 cell monolayers were wounded and incubated for either 4 hours (T84) or 20 hours (HT29-D4 and CaCO2/TC7) with or without *Sb* supernatant. Results are expressed as the percentage of cell migration compared to control. Data represent the mean ± SD of 3 separate experiments. ** *P*<.01.

To investigate any direct effect of *Sb* supernatant on cell restitution, we used HCT-8/E11 cells for their wound-filling capacity [25]. *In vitro*, *Sb* supernatant markedly enhanced wound repair (Figure **1A**) in a dose-dependent manner (not shown) with a maximal effect obtained with a 1/8 dilution. We therefore used this dilution in subsequent experiments. Time lapse videomicroscopy experiments (see supplemental video **S1** and **S2)** revealed that cells treated with *Sb* supernatant exhibited a higher rate of migration than cells treated with a unused culture medium for *Sb* (80 µm/h versus 40 µm/h). Quantification of the surface area recovered by the cells indicated that *Sb* supernatant exerted its effect as early as 1 hour after wounding and persisted for up to 5 hours (Figure **1B**).

The motogenic effect of *Sb* supernatant was quite similar to that observed using type I insulin-like growth factor (IGF-I), a well known inductor of cell restitution (Figure **1C**) [24]. To confirm the specificity of these observations, we evaluated the impact of *Sc* supernatant on cell migration. As illustrated in Figure **1C**, the supernatant of *Sc* did not modulate wound repair. Furthermore, similar results as those obtained with *Sb* supernatants in HCT-8/E11 cells were observed using other polarized intestinal cell lines including HT29-D4, T84 and CaCo2/TC7 (Figure **1D**), indicating that the observed phenomenon could be common to all intestinal cell lines. We verified by flow cytometry analysis that T84 cells express both α2β1and αv integrins indicating that the reduced effect of *Sb* in this cell line was not associated with a lack of integrin expression (not shown).

### *Sb*-induced cell restitution is independent of cell proliferation

**Figure 2:**
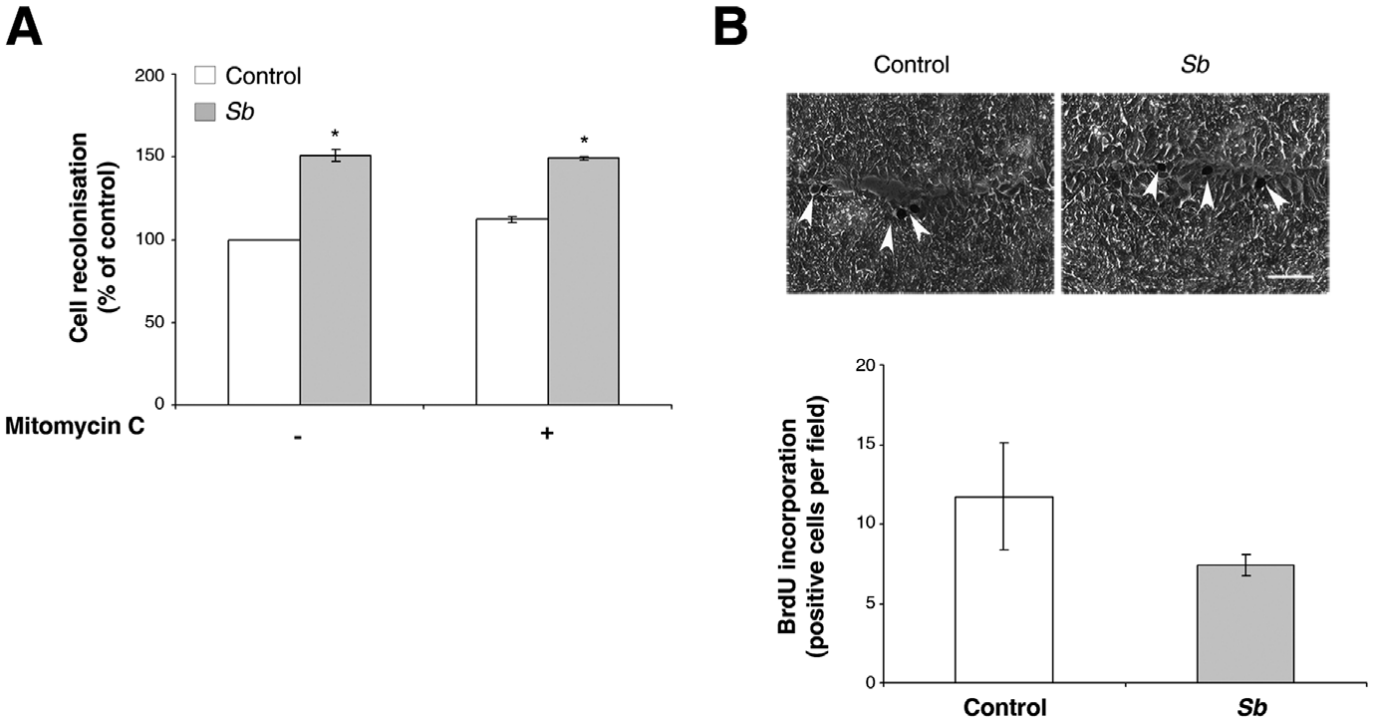
*Sb*-stimulated cell restitution is independent of cell proliferation. (**A**) HCT-8/E11 cell monolayers were incubated for 2 hours with (+) or without (−) 2.5 µg/mL mitomycin C, wounded and cultured for an additional 5 hours period with or without mitomycin C, in the absence or presence of *Sb* supernatant. Wound closure was evaluated as described in Methods. Results are expressed as the percentage of cell migration compared to control. Data represent the mean ± SD of 3 separate experiments. * *P*<.05. (**B**) Confluent monolayers were wounded and incubated with or without *Sb* supernatant for 4 hours. BrdU (10 µmol/L) was added and incubation continued for an additional hour. Cells that had incorporated BrdU into their DNA were detected as described in Methods and are indicated by arrowheads. Data shown are from a representative experiment out of 3 performed. Scale bar: 300 µm.

To determine whether the enhanced wound repair observed in response to *Sb* supernatant was caused by an increase in cell migration and/or cell proliferation, HCT-8/E11 cells were treated with 2.5 µg/ml mitomycin C, an inhibitor of DNA replication, both before and during wound healing assays. Mitomycin C did not attenuate the *Sb*-dependent wound repair, indicating that *Sb* acts by increasing epithelial cell migration with no effect on proliferation (Figure **2A**). Furthermore, as a marker of cell proliferation rate, the extent of BrdU incorporation into cells was measured. As shown in Figure **2B**, few nuclei of migrating cells exhibited uptake of BrdU. Moreover, no significant difference between untreated or *Sb*-treated cells was observed. Taken together, these findings indicate that *Sb* supernatant improves intestinal epithelial restitution independently of cell proliferation.

### *Sb* supernatant improves enterocyte crypt-villus migration

**Figure 3:**
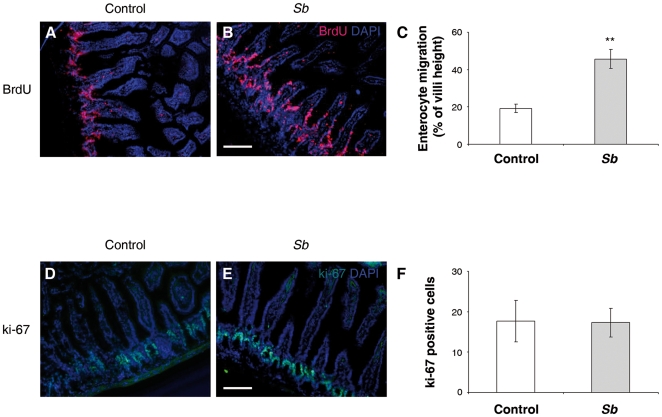
*Sb* improves mouse enterocyte migration along the crypt-villus axis without affecting crypt cell proliferation. Control and force-fed *Sb* mice were injected with BrdU 24 hours before sacrifice. Small intestinal tissues were processed for immunostaining. The BrdU-labeled enterocyte migration along the crypt-villus axis was traced on tissue sections by immunostaining with an anti-BrdU Ab (**A** and **B**) and quantitatively assessed (**C**) as described in Methods. (n = 5). ** *P*<.01. The proliferative marker ki-67 was detected in small intestine of control (**D**) or *Sb* force-fed mice (**E**) and the number of ki-67 positive cells per crypt were evaluated (**F**) (n = 5). Scale bar: 150 µm.

To evaluate whether *Sb* supernatant regulates enterocyte migration *in vivo*, C57BL6J mice were force-fed with PBS solution with or without 1 mg/ml *Sb*, daily, for 1 week. This treatment did not modify villus length (not shown). BrdU was administrated to control and *Sb* force-fed mice 24 hours before death. This strategy allowed the calculation of the rate, and extent to which BrdU-labelled cells migrated from the crypts to the tips of the villi [26]. In control animals, BrdU-stained cells were detected mainly within the crypts (Figure **3A**). By contrast, in *Sb* force-fed mice, BrdU positive cells were distributed from the crypt to the tips of the villi (Figure **3B**). Statistical analysis revealed that *Sb* significantly enhanced cell migration along the crypt-villus axis (migration to 45% of villus height in *Sb* treated animals, compared to 20% of villus height in controls) (Figure **3C**).

To ensure that *Sb* did not promote the expansion of the proliferative compartment, ki-67, a marker of proliferating cells, was immunolocalized. As depicted in Figures **3D and 3E**, ki-67 positive cells were detected only within the crypts in both control and *Sb* force-fed mice. Moreover, *Sb* treatment did not modulate the number of proliferating cells in the crypts (Figure **3F**). These finding indicate that *Sb* could modulate enterocyte migration, both *in vitro* and *in vivo*, without affecting the proliferative compartment.

**Figure 4:**
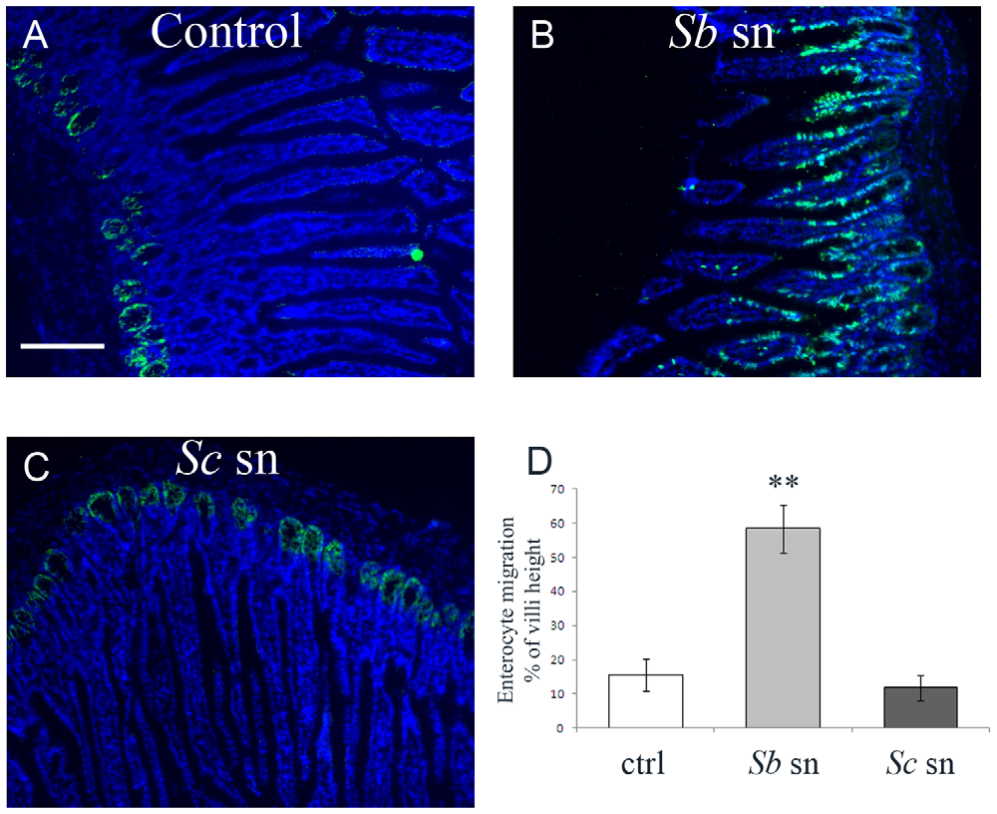
*Sb* supernatant but not *Sc* supernatant improves mouse enterocyte migration along the crypt-villus axis. Mice were daily force-fed for one week with unused culture medium for *Sb* (control), *Sb* supernatant (*Sb* sn) or *Sc* supernatant (*Sc* sn) then injected with BrdU 24 hours before sacrifice. Small intestinal tissues were processed for immunostaining. The BrdU-labeled enterocyte migration along the crypt-villus axis was traced on tissue sections by immunostaining with an anti-BrdU Ab (**A**, **B and C**) and quantitatively assessed (**D**) as described in Methods. (n = 5). ** *P*<.01. Arrow: basal site of the enterocytes. Scale bar: 150 µm.

To compare the effect of the whole yeast and the yeast supernatant, we daily administrated to the mice for one week, either the whole yeast either the culture supernatant of *S.boulardii*. A group of mice were also force-fed daily with supernatant of *S. Cerevisiae*. As depicted on Figures **4A and 4C**, BrdU-stained cells were detected mainly within the crypts in both control and *Sc* supernatant force-fed mice. However, in *Sb* supernatant force-fed mice, BrdU positive cells were distributed from the crypt to the tips of the villi (Figures **4B and 4D**). These data suggest that *Sb* supernatant, but not *Sc* supernatant, contains molecule(s) that induce migration of enterocytes *in vivo*.

### α2β1 integrin collagen receptor is involved in *Sb*-dependent cell restitution

**Figure 5:**
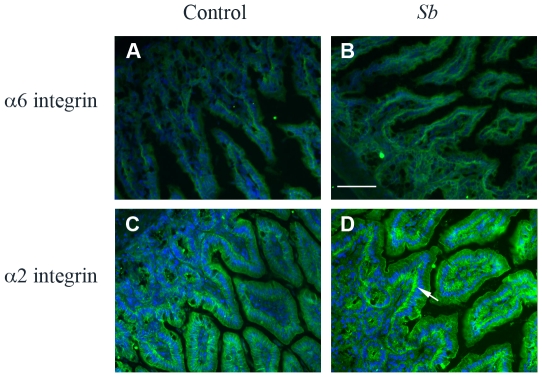
α2β1 integrin is relocalized in the intestine of *Sb* force-fed mice. Intestinal tissues from control (**A** and **C**) or *Sb* force-fed mice (**B** and **D**) were immunostained with Abs against α6 (**A** and **B**) or α2 integrin (**C** and **D**). Each image is a representative image taken from tissue sections of five mice. Scale bar: 130 µm.

Enterocyte migration requires dynamic interactions between ECM molecules, such as laminin isoforms and collagens, and their cell surface receptors [27]. Therefore we determined whether *Sb* affected the distribution of α2 and α6 integrin subunits, receptors of collagens and laminin, respectively. In both control and *Sb* force-fed mice, α6 was detected at the basolateral surfaces of enterocytes on the villus and in the crypt (Figure **5A and 5B**). In control mice, α2 integrin was found mainly at the lateral sites of the enterocytes whereas it was significantly redistributed to the basal sites in *Sb* force-fed mouse cells (Figure **5C and 5D**). These results suggest that α2 integrin participates in *Sb*-enhanced enterocyte migration.

**Figure 6:**
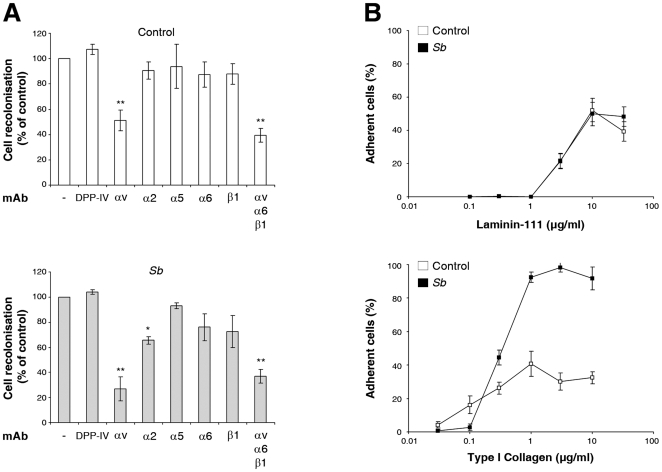
*Sb*-stimulated cell restitution requires modulation of the interaction between α2β1 integrin and collagen. (**A**) HCT-8/E11 cell monolayers were wounded and incubated with or without *Sb* supernatant. The monolayers were further incubated with or without (−) function-blocking anti-integrin mAbs (10 µg/mL) 1 hour before wounding and during cell migration. Wound closure was determined as described in Methods. Results are expressed as the percentage of cell migration compared to control. Data represent the mean ± SD of 5 separate experiments. ** *P*<.01; * *P*<.05. A mouse anti-DPP IV (DPPIV) mAb was used as a control for anti-integrin mAb specificity. (**B**) Isolated HCT-8/E11 cells were treated with or without *Sb* supernatant and plated on either laminin-111 or type I collagen at the indicated concentrations. Cell-ECM adhesion was evaluated as described in Methods. Results are expressed as the percentage of cell adhesion. Data represent the mean ± SD of 3 separate experiments.

To confirm the role of α2β1integrin in *Sb*-enhanced enterocyte migration, HCT-8/E11 cells, which express the integrins α2β1, α3β1, α5β1, αvβ5, and α6β4 (unpublished data), were incubated with function blocking anti-integrin mAbs during cell migration. As observed in Figure **6A**, in control cells only the anti-αv integrin mAb inhibited the capacity of untreated HCT-8/E11 cells to migrate by approximately 50%. Interestingly, *Sb*-dependent cell migration was also inhibited by the anti-αv mAb (70%), and additionally with the anti-α2 (40%) mAb. Altogether these data indicate that the *Sb*-induced enterocyte migration depends on α2β1 integrin. It should be noted that incubation with a mixture containing anti-αv, -α6 and -β1 integrin mAbs did not totally abolish cell migration in either condition, suggesting the participation of integrin-independent cell adhesion molecules in this process.

### *Sb* modulates α2β1 integrin-mediated adhesive properties

**Figure 7:**
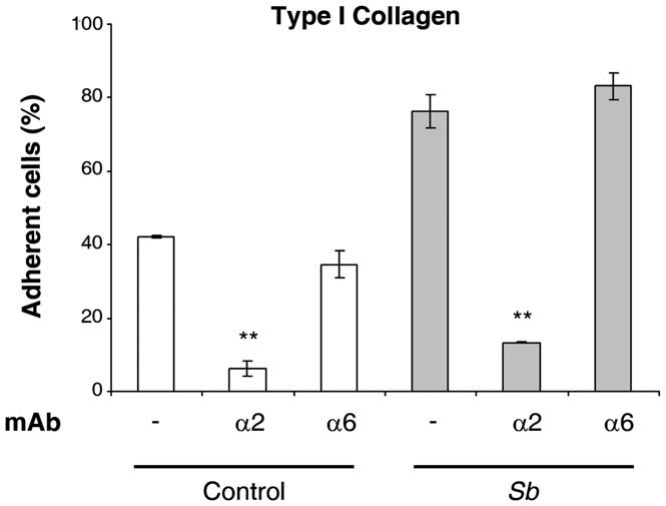
α2β1 integrin is the sole receptor for type I collagen in HCT8/E11 cells. Isolated HCT-8/E11 cells were incubated with or without *Sb* supernatant in the absence (−) or presence of either anti-α2 or -α6 integrin mAbs. Cells were then seeded on type I collagen (3 µg/ml). Cell adhesion was evaluated as described in Methods. Data represent the mean ± SD of 3 separate experiments. ** *P*<.01.

As adhesion is essential for cell migration, we next examined whether treatment with *Sb* supernatant could alter HCT-8/E11 cell attachment to ECM proteins. Cells incubated with or without *Sb* supernatant were allowed to attach to increasing concentrations of purified laminin-111, or type I collagen. HCT-8/E11 cells attached to both matrices (Figure **6B**). *Sb* supernatant did not alter cell adhesion to laminin-111. However, it dramatically increased the percentage of adherent cells on the type I collagen matrix. Adhesion assays performed in the presence of anti-integrin function blocking mAbs revealed that α2β1 integrin is the sole receptor for type I collagen in these cells (Figure **7**). Thus, the increase in cell adhesion to type I collagen upon *Sb* treatment most likely occurs through the modulation of the α2β1 integrin functional state.

### *Sb* supernatant increases α2β1 integrin activation state

**Figure 8:**
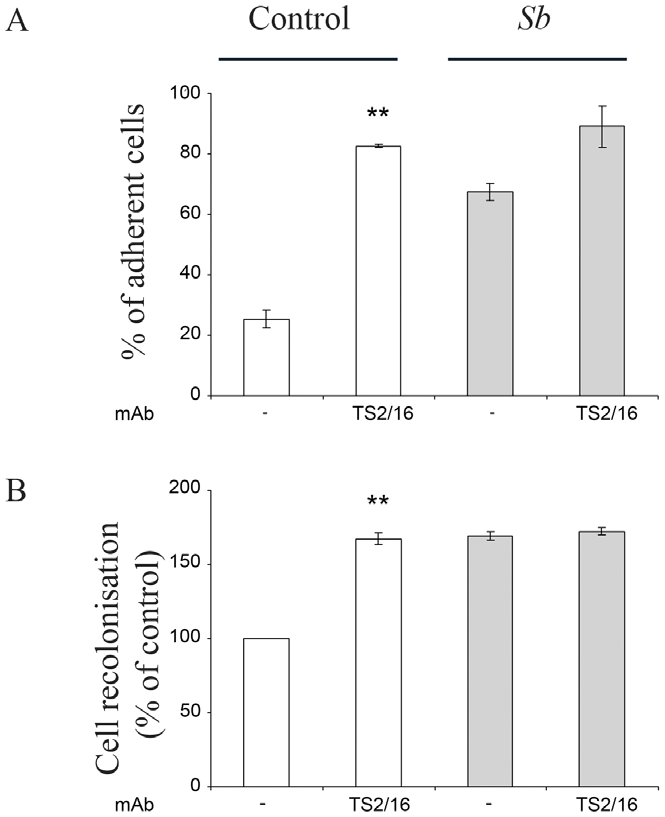
*Sb* modulates integrin activation state. HCT-8/E11 cells were incubated with or without *Sb* supernatant in the absence (−) or the presence of a β1 integrin-activating mAb (TS2/16) (**A**). Cells were allowed to adhere to 3 µg/ml type I collagen for 30 minutes. Cell adhesion was evaluated as described in Methods. ** *P*<.01. (**B**) Cells were allowed to migrate as described in Methods. Results are expressed as the percentage of cell migration compared to control. Data represent the mean ± SD of 3 separate experiments. ** *P*<.01.

We postulated that *Sb* supernatant exerts its effects by activating the α2β1 integrin. To test such a possibility, we activated the α2β1 integrin using TS2/16, a β1 integrin activating mAb [28]. As expected, α2β1 activation increased cell adhesion onto type I collagen (Figure **8A**). This activation also led to an increase in cell migration such as that observed with *Sb* supernatant (Figure **8B**). Pretreatment of cells with integrin α2β1 activating factor improved neither *Sb*-stimulated cell adhesion nor cell restitution (Figure **8A and 8B**), strongly suggesting that *Sb* secretes factors that activate α2β1 integrin, either directly or indirectly.

### *Sb* regulates the α2β1 integrin signaling pathway

**Figure 9:**
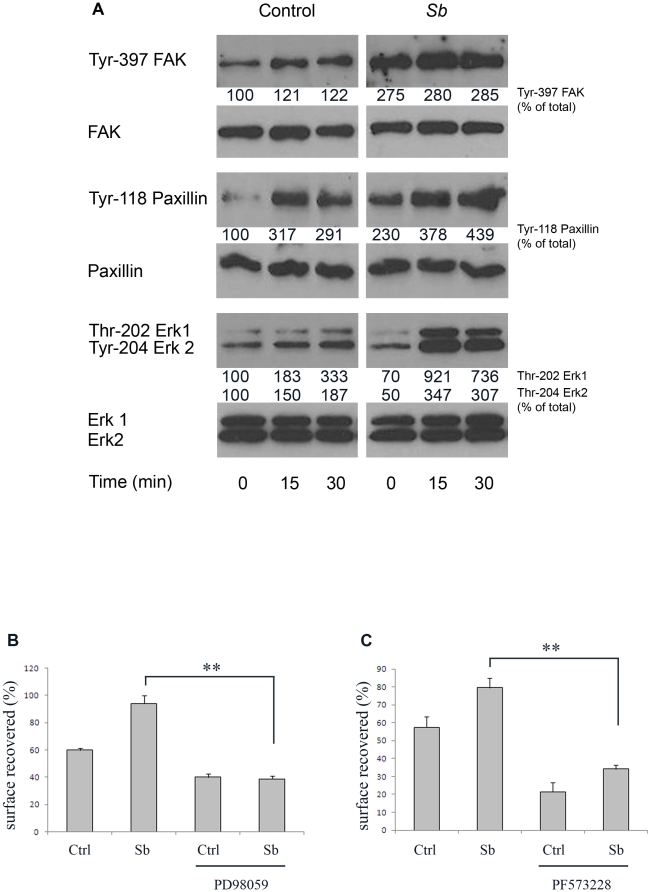
*Sb* activates FAK, paxillin and MAPK. (**A**) HCT-8/E11 cells were allowed to adhere on type I collagen after *Sb* pretreatment. The phosphorylation of tyrosine residues in FAK (Tyr-397 FAK), paxillin (Tyr-118 paxillin) and Thr-202/Tyr-204 in MAPK (Erk1 and Erk2) was determined after cell lysis at the indicated times of cell adhesion. Samples were analyzed by western blot analysis. Equal amounts of protein were analyzed and loading amounts were verified by probing the blot with anti-FAK, anti-paxillin or anti-Erk1/Erk2 Abs. The intensity of the bands was measured after scanning of the autoradiograms. Values presented on the blots correspond to percentage of band intensity compared to control. (**B** and **C**) HCT-8/E11 cell monolayers were wounded and incubated with or without *Sb* supernatant. The monolayers were further incubated with or without PD 98059, a MAPK inhibitor (**B**) or with or without PF 573228, a FAK inhibitor (**C**), 1 hour before wounding and during cell migration. Wound closure was determined as described in Methods. Results are expressed as the percentage of wound repair. Data represent the mean+SD of 3 separate experiments. ** *P*<.01.

After activation, integrins quickly associate within the actin cytoskeleton and cluster together to form focal adhesions, multimolecular complexes that contain signaling molecules including focal adhesion kinase (FAK), paxillin and Erk1/2. Activation of these signaling molecules leads to the modulation of cell migration. We therefore checked the phosphorylation status of these different key signaling molecules. As observed in Figure **9A**, cell adhesion to type I collagen promoted phosphorylation of both FAK and paxillin on the Y397 and Y118 tyrosine residues respectively. Interestingly, *Sb* supernatant increased the tyrosine phosphorylation level of both proteins. *Sb* also upregulated the tyrosine phosphorylation status of Erk1/2, another protein associated with integrin signaling pathways (Figure **9A**). We next tested the effect on cell migration of PD 98059 and PF 573228, inhibitors of MAPK and FAK respectively. As observed on Figures **9B and 9C**, these inhibitors fully blocked the *Sb*-induced cell migration. These results confirm that *Sb* stimulates cell migration through the activation of multiple signaling pathways including activation of FAK and Erk1/2.

**Figure 10:**
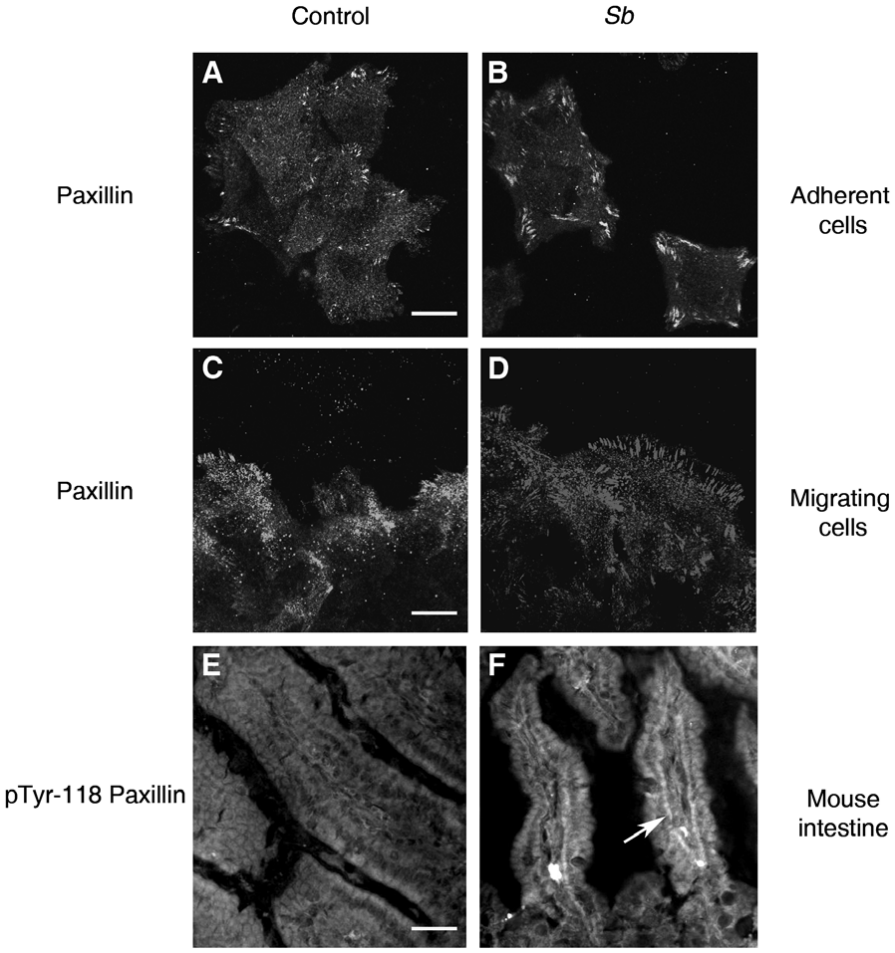
*Sb* modulates integrin signaling pathway both *in vitro* and *in vivo*. HCT-8/E11 cells were pretreated (**B**) or not (**A**) for 2 hours with *Sb* supernatant, and then allowed to adhere to type I collagen-coated surfaces (3 µg/ml) for 30 minutes. After fixation and permeabilization, cells were stained with an anti-paxillin Ab, the binding of which was revealed with an AlexaFluor 488-conjugated anti-mouse secondary Ab. Scale bar: 20 µm. HCT-8/E11 cell monolayers were wounded and incubated without (**C**) or with (**D**) *Sb* supernatant for 5 hours. Paxilin was revealed using a specific anti-paxillin Ab. Scale bar: 20 µm. Intestinal tissues from control (**E**) or *Sb* force-fed mice (**F**) were immunostained with an Ab against Tyr-118 paxillin. Stainings were analyzed using a Leica confocal microscope. Scale bar: 100 µm.

We analyzed the impact of *Sb* supernatant on the organization of adherence structures (Figure **10**). Paxillin staining revealed that control cells, plated on type 1 collagen, exhibited few focal adhesion structures. However paxillin staining in *Sb* treated cells displayed more focal adhesion structures that appeared to be thicker than those observed in the control condition (Figure **10A and 10B**). Staining of focal adhesion structures on migrating cells yielded a similar pattern (Figure **10C and 10D**). This indicates that treatment with *Sb* supernatant is associated with functional and structural modifications of focal adhesion structures.

To explore whether *Sb* also regulates focal adhesion structures *in vivo*, we analyzed the paxillin tyrosine phosphorylation status on intestine from mice force-fed with PBS alone, or PBS containing 1 mg/ml lyophilized *Sb*. As depicted in Figure **10E**, an anti-Y118 paxillin Ab failed to stain any structure in control C57BL6J mice. However, in *Sb* force-fed mice, staining was detected, confined to the basal surface of the enterocytes (Figure **10F**). This strongly suggests that *Sb* promotes the activation of adhesion structures found in enterocytes *in vivo*.

Taken together these data indicate that *Sb* secretes factors that strengthen α2β1/collagen interactions leading to enhanced outside-in signaling and focal adhesion structure reorganization. This was associated to an increase in cell migration.

## Discussion

Besides a marked infiltration of inflammatory cells, epithelial cell damage is also frequently observed in the mucosal lesions of IBD such as ulcerative colitis, Crohn's disease or in infectious gastroenteritis [2]. Therefore, complete remission of such diseases requires both the cessation of inflammation and the repair of damaged epithelium. The development of novel therapies that accelerate the repair of intestinal epithelium has recently begun and various molecules are now being considered for clinical use including epidermal growth factor in combination with mesalamine [29], keratinocyte growth factor [30], and hepatocyte growth factor [31]. However, a better understanding of the biological effects of these molecules must be ascertained, not least to identify any undesired secondary effects, such as tumorigenesis.

In the present study, we analyzed the effect of *Sb* on the capacity of intestinal epithelial cells to reseal a wound. According to our findings, the impact of *Sb* on epithelial cells can be summarized as follows: (1) *Sb* supernatant enhances the restitution of various differentiated human intestinal epithelial cell lines *in vitro*; (2) in the mouse intestine, *Sb* improves migration of enterocytes along the crypt-villus axis; (3) *Sb* does not influence enterocyte proliferation either *in vitro* or *in vivo*; (4) *Sb* stimulates α2β1 integrin, thus activating molecules such as FAK, paxillin and ERK1/2, which are associated with integrin signaling pathways and which modulate cell-type I collagen I interaction; (5) concomitant with this activation, *Sb* modulates both the structure and the activity of focal adhesions. Taken together, these effects could contribute to the enhancement of intestinal cell restitution.

Intestinal wound healing is initiated by the migration of epithelial cells adjacent to the injured surface into the wound to cover the denuded area. This process, termed epithelial restitution, occurs within a period of minutes to hours and does not require cell proliferation [2]. Through the use of a relevant *in vitro* model [32], we have shown that *Sb* supernatant increased the capacity of differentiated enterocytes to recolonize a wound. The effect of *Sb* on wound healing was associated with an increase in cell motility rather than a stimulation of cell proliferation. Indeed, wound repair was unaffected by treatment with DNA synthesis inhibitors such as mitomycin C and BrdU incorporation experiments indicated that only a few proliferating cells were found in wounded areas. Also *Sb* did not alter cell growth (not shown). Furthermore, videomicroscopy experiments showed that *Sb* supernatant doubled the migration rate of HCT-8/E11 cells and *Sb* increased the rate of migration of enterocytes along the crypt-villus axis in mice without affecting the proliferative compartment. According to these data, we postulated that *Sb* supernatant contains motogenic factor(s) that improve intestinal cell restitution.

Although the physiological pathways involved in intestinal restitution remain partially unexplained, it has been proposed that changes in cell-ECM interactions occur spatially and temporally during this process. Studies carried out on gut tissue revealed that ECM proteins including fibronectin, type I collagen and laminins play a crucial role in regulating cell restitution [6], [7], [33]. They have also demonstrated the requirement for the binding and interaction of ECM-integrins such as α3β1, α6β1, α6β4 (laminins receptors), α2β1 (collagens receptor), or αvβ3 or αvβ6 (fibronectin receptors) for intestinal restitution [6], [34]. In line with these results, we have provided evidence for the requirement of α2β1 integrin facilitated cell-ECM interaction for *Sb*-induced restitution. *Sb* was shown to promote the redistribution of the collagen receptor from the lateral to the basal membrane domain of enterocytes. Inhibition assays using anti-integrin mAbs demonstrated that α2β1 integrins participate in the *Sb*-induced cell migration, suggesting that collagens support this process. Also, adhesion assays suggested that *Sb* specifically modulates the α2β1-dependent cell interaction with type I collagen.

The mechanisms by which *Sb* modulates integrin-ECM interactions and subsequent intestinal restitution need to be explored in more depth. However, at least two possibilities exist. Firstly, cell restitution could be modulated through the regulation of the integrin functional state. In accordance with this, some data have highlighted dynamic changes in integrin affinities for ligands. These changes are thought to occur in response to extracellular cues, and can modify cell migration [35]. In line with these data, we demonstrated that factors secreted by *Sb* can activate α2β1integrin. Indeed, cell treatment with the β1 integrin activating factor TS2/16 mAb [28], [36] promoted the same increase in cell adhesion as *Sb*. Moreover, cell pretreatment with TS2/16 mAb did not enhance the impact of *Sb* on cell adhesion or cell restitution. In addition, *Sb* did increase α2β1 integrin signaling pathways.

*Sb* has been shown to generate transduction pathways, including activation of Erk1 and Erk2 in intestinal mucosal cells [11]. In the present study, we demonstrated that *Sb* can modulate pathways leading to cell migration. A large number of molecules, including FAK, have been suggested as candidates for recruitment in adhesion structures upon integrin activation. Furthermore these molecules may serve as conduits for the transmission of the force that is necessary for cell migration and for bidirectional signaling between the cell interior and its environment [37]. FAK activation leads to the stimulation of other signaling proteins such as paxillin, thereby activating various signaling pathways crucial in the regulation of cell adhesion and migration [37]. In the present study, we demonstrated that *Sb* secretes factor(s) that alter the FAK/paxillin signaling pathways. In mice, relocalization of α2β1 integrin to the basal domain of the enterocyte following *Sb* treatment is associated to paxillin phosphorylation of the Y118 residue of the same domain. Also *Sb* increased the tyrosine phosphorylation levels of FAK, paxillin and Erk1/2. Furthermore, *Sb*-treated cells exhibited focal structures that appeared thicker when compared to control cells. These observations clearly indicate that cell treatment with *Sb* resulted in a strengthening of α2β1/collagen interactions as shown by enhanced outside-in signaling and focal adhesion reorganization.

*Sb* has been proposed to act as a shuttle able to liberate at least 1500 as yet not fully characterized molecules during intestinal transit [38]. This large number of secreted peptidic and non-peptidic factors may at least partially explain why *Sb* has pleiotropic effects on intestinal mucosa and therapeutic effects on such a wide variety of gastrointestinal disorders [9], [38], [39]. Some of the yet unidentified molecules may interfere with host cell signaling pathways and thereby modulate host cell behavior [38]. Indeed, *Sb* has been shown to secrete polyamines that are implicated in intestinal cell maturation, enzyme expression and membrane transport mechanisms [38]. In addition, *Sb* produces other factors that reduce inflammation by blocking NF-κB activation [21], [40] and enhancing PPAR-γ expression [41]. The motogenic molecule(s) secreted by *Sb* remain to be elucidated. However, our preliminary data suggest that the motogenic factor(s) secreted by *Sb* is (are) an heat instable molecule(s) (result not shown). Further studies are now needed to explore the molecular mechanisms by which supernatant of *Sb* alters cell migration.

In conclusion, this report demonstrates for the first time that *Sb* secretes motogenic factors that can improve intestinal restitution. These factors exerted their effects through dynamic fine regulation of integrin-mediated adhesion to the ECM. This could be of major importance in the future treatment of diseases characterized by severe mucosal injury, such as IBD or infectious diarrhea and colitis.

## Supporting Information

Video S1 HCT-8/E11 cell monolayers were wounded as described in Methods, then placed in a temperature and CO_2_-controlled chamber mounted on a Nikon TE2000 inverted microscope. Images were captured every 5 minutes for a total observation duration of 5 hours using a Cool SnapHQ camera (Princeton Instrument) through a 10× objective lens.
(MP4)

Video S2 HCT-8/E11 cell monolayers were wounded as described in Methods and incubated with *Sb* supernatant. Plates were placed in a temperature and CO_2_-controlled chamber mounted on a Nikon TE2000 inverted microscope. Images were captured every 5 minutes for a total observation time of 5 hours using a Cool SnapHQ camera (Princeton Instrument) through a 10× objective lens.
(MP4)
